# Structure- and Content-Dependent Efficiency of Cas9-Assisted DNA Cleavage in Genome-Editing Systems

**DOI:** 10.3390/ijms232213889

**Published:** 2022-11-11

**Authors:** Svetlana V. Baranova, Polina V. Zhdanova, Alexander A. Lomzov, Vladimir V. Koval, Alexander A. Chernonosov

**Affiliations:** 1 Institute of Chemical Biology and Fundamental Medicine, Siberian Branch of Russian Academy of Sciences (ICBFM SB RAS), 630090 Novosibirsk, Russia; 2 Department of Natural Sciences, Novosibirsk State University, 630090 Novosibirsk, Russia

**Keywords:** CRISPR-Cas system, Cas9 activity, oligonucleotide mismatch, cleavage, thermodynamics

## Abstract

Genome-editing systems, being some of the key tools of molecular biologists, represent a reasonable hope for progress in the field of personalized medicine. A major problem with such systems is their nonideal accuracy and insufficient selectivity. The selectivity of CRISPR-Cas9 systems can be improved in several ways. One efficient way is the proper selection of the consensus sequence of the DNA to be cleaved. In the present work, we attempted to evaluate the effect of formed non-Watson–Crick pairs in a DNA duplex on the efficiency of DNA cleavage in terms of the influence of the structure of the formed partially complementary pairs. We also studied the effect of the location of such pairs in DNA relative to the PAM (protospacer-adjacent motif) on the cleavage efficiency. We believe that the stabilization of the Cas9-sgRNA complex with a DNA substrate containing noncomplementary pairs is due to loop reorganization in the RuvC domain of the enzyme. In addition, PAM-proximal mismatches in the DNA substrate lower enzyme efficiency because the “seed” region is involved in binding and cleavage, whereas PAM-distal mismatches have no significant impact on target DNA cleavage. Our data suggest that in the case of short duplexes with mismatches, the stages of recognition and binding of dsDNA substrates by the enzyme determine the reaction rate and time rather than the thermodynamic parameters affected by the “unwinding” of DNA. The results will provide a theoretical basis for predicting the efficiency and accuracy of CRISPR-Cas9 systems at cleaving target DNA.

## 1. Introduction

The genome-editing system CRISPR-Cas9 is one of the most active research topics in biology and biochemistry in recent years. In this system, the Cas9 protein performs a targeted search and makes a double-stranded break in a DNA molecule [1]. Cas9 is a 1368-amino-acid multidomain DNA endonuclease that has two nuclease domains: an HNH-like domain that cleaves the DNA strand complementary to the guide RNA sequence (target strand) and a RuvC-like nuclease domain responsible for cutting the DNA strand opposite the complementary strand [2,3,4].

The Cas9 nuclease is programmed by CRISPR-RNA (crRNA), which carries out specific recognition of the target sequence [1]. As a rule, crRNA acts in tandem with tracrRNA, which is responsible for binding the enzyme [5]. A guide RNA contains both crRNA and tracrRNA in a single molecule. Such a combined molecule of ~120 nucleotides is called a single guide RNA (sgRNA) [6]. The conserved region (100-nucleotide strand) that forms the specific spatial structure that the Cas9 enzyme recognizes is located at the 3′ end of sgRNA [7]. In the first step, RNA binds to the protein and forms the Cas9-sgRNA complex [2]. The sequence (~20 nucleotides) responsible for binding to DNA is located at the 5′ end of sgRNA [7]. DNA incision occurs only if a PAM (protospacer-adjacent motif) is located downstream of the complementary RNA sequence. The protospacer is a trinucleotide of the NGG type, where N can be any nucleotide in the DNA. The incision occurs between the third and fourth nucleotide from the PAM sequence [3,7].

The CRISPR-Cas9 genome-editing system is a promising tool for genetic engineering and therapeutic applications [8]. A major problem with the CRISPR-Cas9 technology is the cleavage of off-target DNA, i.e., a target with a sequence incompletely complementary to sgRNA [1,5]. The enhancement of enzyme specificity can be achieved in various ways. One of them is to create mutants of the Cas9 protein [8,9]. For example, mutants with high fidelity (Cas9-HF1: N467A, R661A, Q695A, and Q926A) or enhanced specificity [eSpCas9(1.1) K848A, K1003A, and R1060A] have been designed [8]. Additionally, the specificity of the Cas9 enzyme is increased by a factor of 100–1500 when it is used in combination with various mutant nickases (Cas9-D10A or Cas9-H840A) which can be targeted to sites on opposite DNA strands at distances of up to 100 bp from each other [9].

On the other hand, by modifying the sgRNA sequence in the “seed” region, researchers can modulate the CRISPR–Cas9 system, direct it to virtually any DNA sequence of interest in the genome, and introduce a specific double-stranded break [2]. In order to determine the sequence of genomic-DNA cleavage sites produced by the Cas9 enzyme, the authors of Reference [10] developed a biochemical method (site-seq) wherein Cas9 is programmed by various sgRNAs. They demonstrated that the number of recognized sites depends upon the sgRNA sequence and the concentration of the Cas9 endonuclease [10]. The use of truncated guide RNAs (17–18 bp), which are more sensitive to nucleotide substitutions, reduces the occurrence of adverse off-target events [9]. Thus, the potential use of CRISPR-Cas9 in genomic engineering holds promise for the treatment of genetic disorders, including many types of cancer and neurodegeneration as well as sickle cell anemia, cystic fibrosis, viral infections, immunological disorders, and other diseases [2].

In order to develop high-efficiency and high-specificity genome-editing tools, it is crucial to study the basic mechanisms by which Cas9 recognizes various mismatches. Investigating the interaction of the Cas9-sgRNA complex with double-stranded DNA is important for understanding the specificity of the CRISPR-Cas9 system. In References [1,5], researchers assessed the effect of mismatches on DNA cleavage by creating noncomplementary pairs through nucleotide substitutions in the DNA sequence of complementary RNA or in sgRNA itself. It is equally important to investigate what effects nucleotide substitutions in another DNA strand exert on substrate recognition and Cas9 enzymatic activity. In this work, we examined how mismatches in a substrate DNA at different positions with respect to the PAM sequence affect this enzyme’s activity. Short duplexes (32 bp) whose sequence fully or partially matched the sgRNA fragment were employed as substrates in this project. We calculated the thermodynamic parameters (energy and melting temperature) of the duplexes corresponding to the target substrate and the duplexes containing noncomplementary pairs.

The calculated thermodynamic parameters and the degree of cleavage of such substrates by the Cas9-sgRNA complex were compared.

## 2. Results and Discussion

Previously, it has been shown that DNA unwinding and subsequent conformational changes in the enzyme affect the activity of HNH and RuvC domains performing catalytic cleavage of the DNA substrate after the binding [1,8,11,12]. Therefore, first, we investigated the impact of mismatches on the thermodynamic characteristics of the analyzed duplexes. The sequences of substrates were chosen so that the mismatches were located in the area directly around the cleavage site near the PAM region (NGG). The PAM region had a GGG motif, which is typical for the Cas9 from Streptococcus pyogenes (spyCas9) used in our experiments.

### 2.1. The Calculation of Thermodynamic Parameters

In order to try to rationalize the differences between the effects of various mismatch positions, we performed molecular dynamics (MD) simulations of nine oligonucleotide duplexes. After the computer simulation, we obtained the thermodynamic parameters of duplexes’ formation by analyzing the trajectories using the Molecular Mechanics Generalized Born Surface Area (MM-GBSA) method (Table 1).

MD simulations began with the generation of oligonucleotide duplex structures using XLEaP as described in Methods. All double-stranded DNA (dsDNA) structures remained stable throughout all MD trajectories, and their terminal nucleotide pairs did not melt.

In order to calculate the interstrand binding energies, we used only the MM-GBSA method, as it is the most accurate (the error for nucleic acids is ~9%) [13]. In the analysis of the formation of each DNA duplex, the trajectories of the components of the duplex were separated from the trajectory of the initial system, and the binding energy of the duplex components was calculated. Depending on the mismatch position and context, energies varied markedly.

For S01 (no substitution), the interaction energy of the two strands was found to be ΔE = −293.7 ± 0.1 kcal/mol. In the case of S03, where a G:A pair forms at position +2 of the PAM sequence, ΔE is −286.3 ± 0.1 kcal/mol. The replacement of C with A at position +5 of the PAM (S05) resulted in an interstrand oligonucleotide binding energy of −281.6 ± 0.1 kcal/mol. The energy of the G:G pair formation at position +1 in relation to PAM in S07 (ΔE = −287.0 ± 0.1 kcal/mol) is close to that in S03 (ΔE = −286.3 ± 0.1 kcal/mol). The binding energy of the two strands of duplex S06 containing C instead of T at position +3 is ΔE = −290.2 ± 0.1 kcal/mol. The energy of −298.4 ± 0.1 kcal/mol for the dsDNA (S09), where the G:T pair forms at position +4 from the PAM sequence, indicates the highest thermodynamic stability of the duplex in comparison with all other duplexes containing a mismatch(es). The data we obtained are consistent with the data of Reference [14], the authors of which showed that the contribution of one mismatch GT to the stability of the duplex helix also depends upon the neighboring bases of the mismatch. GT mismatches form stable pairs linked by hydrogen bonds, and the stability of duplexes also depends upon neighboring mismatch bases [14].

We obtained an energy of ΔE = −281.8 ± 0.1 kcal/mol for the mismatch located upstream (−2) of the PAM sequence in S02. In this duplex, the resulting C:C pair destabilizes the double helix quite strongly, as evidenced by the lowest energy.

Oligonucleotide duplexes containing two mismatches at different positions were found to bind with similar energy values. For instance, S04, where the mismatches are located on either side of the AT cleavage sequence (G at +2 and A at +5), has an energy of ΔE = −286.6 ± 0.1 kcal/mol. The observed energy for S08, which contains the inverted AT cleavage region (TA), is ΔE = −288.2 ± 0.1 kcal/mol.

### 2.2. Determination of the Melting Point of dsDNAs

Duplexes formed by fully complementary sequences and duplexes containing mismatches were studied by thermal denaturation of DNA. as the melting temperature depends on the pH and ionic strength of a solution [15], the measurement was performed in a buffer that was the closest in composition to the buffer in which the Cas9-sgRNA complex cleaves DNA substrates: pH 6.4, 125 mM KCl, and 6 mM MgCl_2_. To record the melting curves (Figure S1), we measured a change in the absorbance of the oligonucleotides at 260 nm as a function of sample temperature. The melting temperatures (T_m_) for all dsDNAs were determined from the melting curves (Table 1). The T_m_ values correspond to the energy state at which half of the dsDNA molecules melt [15]. It is known that the melting temperature depends upon the composition of an oligonucleotide. GC-rich substrates have the highest temperature [16]. One or more mismatches in the pairing results in a decrease in the melting temperature. Thus, the fully complementary substrate (S01) has the highest melting temperature. The presence of mismatches led to an insignificant decrease in the melting temperature (Table 1).

The authors of Reference [17] have revealed that the stability of duplexes containing noncomplementary base pairs decreases according to certain rules. It depends on both stacking interactions and the formation of hydrogen bonds between the bases. The stability can be ranked depending on the stacking interactions (GG > AG > AA > GT > GC > AT > AC > TT > CT > CC) or depending on the ability to form hydrogen bonds (GC > AG > AT > AA > CT > GG > CC > AC ≈ TT > GT) [16,17]. Guanidine also engages in strong base pairing with noncomplementary bases, and therefore the presence of noncomplementary G:G and G:T pairs in the duplex causes a slight decrease in the melting temperature (S07 and S09; Table 1) compared with the original substrate (S01; Table 1). On the other hand, cytosine forms unstable pairs with noncomplementary bases (mismatch C:C in S02; Table 1), but the melting point does not change significantly either. This finding suggests that the stability of the duplex also depends upon the neighboring bases of the mismatch [18,19]. Duplexes with two noncomplementary pairs (S04 and S08) have the lowest melting point, as expected.

### 2.3. Suitable dsDNA Cleavage Conditions

The Cas9-sgRNA complex performs site-specific hydrolysis of the phosphodiester bond in double-stranded DNA [2,3,20]. As a rule, oligonucleotides of various lengths are utilized as model substrates [4,8,12,20,21,22,23,24,25,26,27]. In the present work, as the model substrates we chose oligonucleotides (32 bp) with nucleotide substitutions in the strand opposite to the single-stranded DNA complementary to RNA, at various positions with respect to the PAM (Table 1). The Cas9-sgRNA complex recognizes DNA through specific interactions with two DNA regions: the protospacer and the sequence adjacent to the PAM. Binding between the target DNA and the complementary sequence of 20 nucleotides at the 5′ end of the sgRNA spacer arises due to base pairing. By contrast, PAM binding is promoted by direct interactions between the amino acid residues of the enzyme and a target DNA [10,12].

A comparison of electron microscopy data with X-ray crystal structures of Cas9 either with or without guide RNA and target DNA revealed conformational reorganization of the two regions toward each other during the nucleic-acid binding [28]. Binding of sgRNA to the enzyme causes the nuclease lobe to turn ~100° relative to the α-helices, resulting in a “gap” where dsDNA binds to sgRNA.

Binding of the target DNA to Cas9 is mediated primarily by contacts with bound nucleic acids rather than direct contacts with the protein [4,21]. After the Cas9-sgRNA complex binds to the target, the enzyme cleaves both DNA strands between the third and fourth nucleotide from the PAM [7]. Cas9 recognizes a sequence with mismatches near the protospacer, and this process can lead to off-target nuclease activity if the PAM is recognized [10]. In order to increase the usability of the CRISPR-Cas9 system, it is necessary to eliminate or minimize the risk of off-target substrate cleavage. For this purpose, it is important to research the efficiency of recognition and cleavage of such off-target substrates. To evaluate the target activity of Cas9 on a plasmid or dsDNA as substrates, researchers use various excesses (50- to 250-fold) of the complex as compared to the substrate [4,20]. The cleavage of substrates containing mismatches proceeds with different efficiency rates. Therefore, to be able to assess the influence of the position of the noncomplementary pair in the oligonucleotide sequence on the efficiency of Cas9, we had to adjust the conditions under which the cleavage of all the substrates presented in Table 1 occurs.

**Figure 1:**
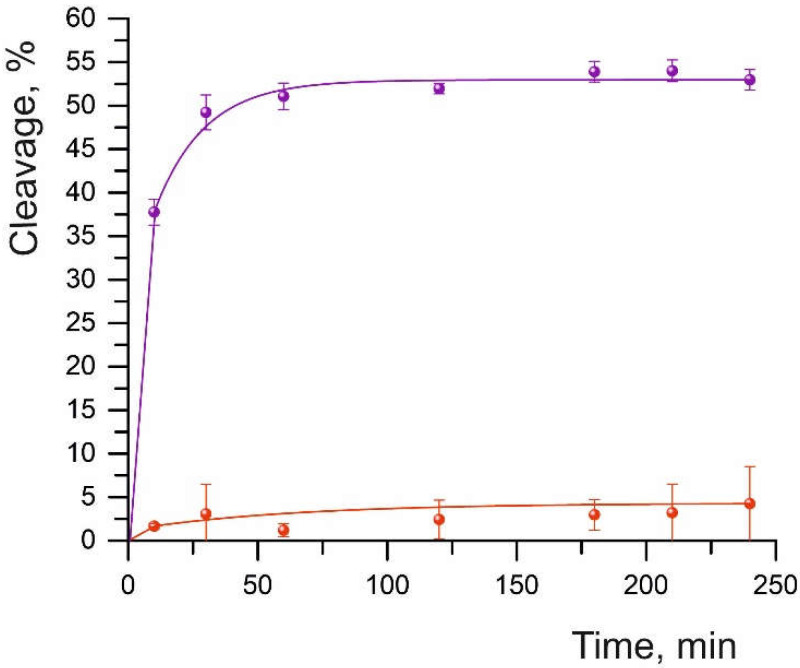
The time curve of the cleavage of substrate S01 (purple curve) and substrate S03 (red curve). The data were averaged from three independent experiments. Sequences of substrates S01 and S03 are presented in Table 1.

**Figure 2:**
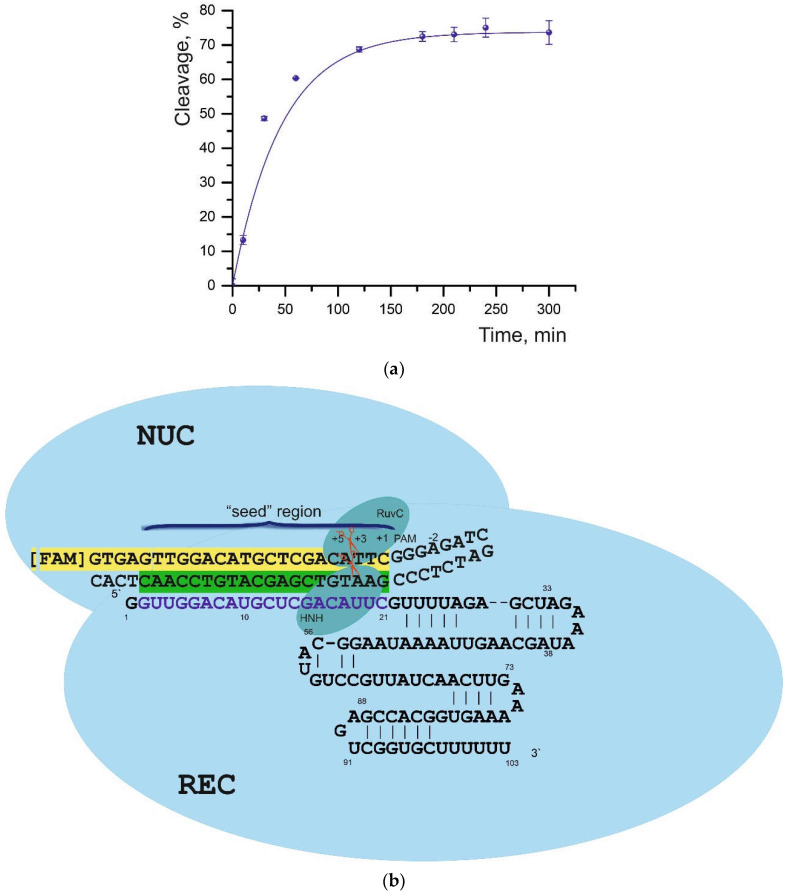
(**a**) The time curve of cleavage of substrate S01. The data were averaged from three independent experiments. The degree (%) of cleavage and error can be found in Table 1. (**b**) Illustration of the complex between Cas9-sgRNA and a DNA substrate. The cleavage site is boldfaced, and sequences forming the complementary duplex are highlighted in green and yellow. REC—recognition lobe of Cas9, NUC—nuclease lobe, which comprises the conserved RuvC and HNH domains.

At an eightfold excess of the Cas9-sgRNA complex over the oligonucleotide substrate, the efficiency of duplex cleavage without mismatches (S01) was 52%. Under these conditions, however, we failed to detect the cleavage of DNA substrates having sequence substitutions at different positions of the DNA/sgRNA complementarity site (Figure 1 and Figure S2). At a ratio of 210:1 (Cas9-sgRNA:dsDNA), the cleavage of all substrates took place successfully (Table 1); therefore, this ratio was chosen for further assays. The maximum cleavage was observed for the S01 substrate: 73% with a half-life of 18.2 min (Figure 2 and Figure S3). The cleavage reaction conditions we selected are consistent with the results of experiments described in Reference [29], where it was shown that the stoichiometry of a target substrate and a ribonucleic complex as well as the cleavage time can be easily adjusted. Those authors observed an increase in reaction efficiency as the concentration of the Cas9-sgRNA complex was raised. Nonetheless, a higher dose of Cas9 improves the efficiency of the cleavage reaction but does not affect the dependence of efficiency on the position of the mismatches [29].

### 2.4. The Efficiency of Cleavage Reactions of the Substrates with Mismatches

As reported by various researchers, the Cas9-sgRNA ribonucleoprotein complex first binds to the neighboring NGG protospacer region and then hybridizes with 8–12 nucleotides of a target (“seed” region) adjacent to the PAM [24,30]. The presence of mismatches in this “seed” region disturbs the assembly of the ribonucleoprotein complex from DNA and the other components [1], thereby possibly reducing cleavage efficiency and prolonging reaction time.

The cleavage rate of substrates containing one or two noncomplementary pairs was found to decrease depending upon the location of the mismatches relative to both the PAM sequence and the cleavage site (Table 1, Figure 2b). The replacement of guanine with cytosine in the distal region (S02 in Table 1, Figure 2b) led to an insignificant decline in the cleavage degree and cleavage rate. Replacement of the first nucleotide from the PAM sequence (cytosine by guanine: S07 in Table 1) yielded approximately the same degree of cleavage but slowed down the reaction rate by half.

This finding is consistent with a study showing that PAM-distal mismatches play an important role in selectivity, DNA unwinding, and conformational dynamics within the enzyme [8], thus leading to longer reaction time.

Replacing thymine with cytosine (S06, Table 1) at the cleavage site not only diminished the degree of cleavage to 10% as compared to S01 but also slowed the reaction rate by half.

**Figure 3:**
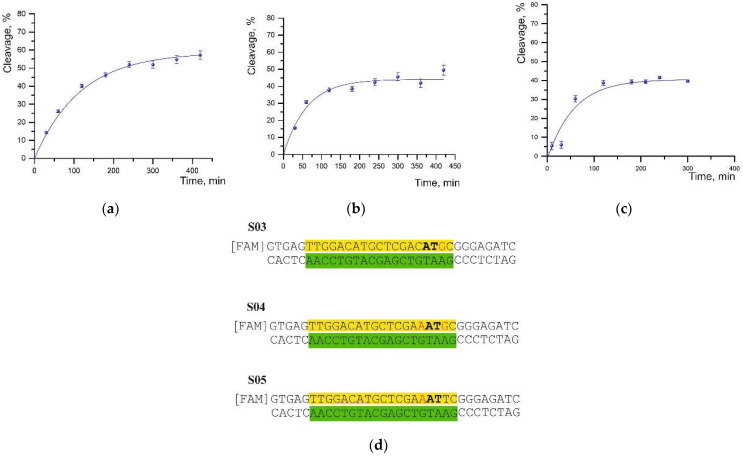
The time curve of the cleavage of substrates (**a**) S03, (**b**) S04, and (**c**) S05. The data were averaged from three independent experiments. The degree (%) of cleavage and error can be found in Table 1. (**d**) The sequences of DNA substrates (S03, S04, and S05). The cleavage site is boldfaced, and the sequences forming a complementary duplex are highlighted in green and yellow.

By contrast, the substitution of thymine with guanine at the position next to the cleavage site in the S03 substrate led to both a lower cleavage degree and a significant (~4-fold) increase in half-cleavage time (Figure 3a,d).

A mismatch at the fifth position of the PAM sequence (cytosine-to-adenine substitution) near the cleavage site in substrates S05 and S04 caused a decrease in the cleavage degree to 44% and 41%, respectively, and an increase in the half-cleavage time (Figure 3b–d).

Moreover, the presence of a second noncomplementary pair in the S04 substrate did not result in significant differences (Table 1). On the contrary, a single substitution of thymine with guanine (S03) led to a more significant increase in half-life but had a weaker effect on the efficiency of cleavage.

A low degree of cleavage was observed for the substrates S09 and S08, which contain a substitution at the cleavage site (Table 1). In the case of the S08 substrate, which contains a double mismatch at the cleavage site, half-cleavage time also increased significantly to 99 min. Most likely, the fourth nucleotide from the PAM sequence is more important for enzyme–substrate complex formation and cleavage than the nucleotide at the third position. The presence of mismatches in the “seed” region tended to significantly reduce the cleavage efficiency. The degree of cleavage in the presence of a single mismatch depends on the position and base type, whereas double mismatches in the target DNA enhance the trends of cleavage efficiency that depend upon the position of the mismatches [29].

Replacement of the first two nucleotides adjacent to the PAM negatively affected Cas9 binding to DNA; therefore, the rate of cleavage of such targets is lower compared to the rate of cleavage of the S01 substrate. This means that Cas9 is sensitive to mismatches at these two positions [5]. Our data are consistent with the theoretical thermodynamic calculations [31], where it was showed that the proximal PAM region of the R-loop structure is more sequence sensitive than the distal PAM region (Table 1, substrates S03–S09).

Cleavage of substrates having a mismatch at position 3–5 relative to the PAM were less effective in comparison with position 2, but the former mismatch is located at the site of DNA substrate cleavage and thus may predominantly affect the catalytic activity of the enzyme [5].

Our data are consistent with results in Reference [32], which indicates that the stabilization of the Cas9-sgRNA complex with a DNA substrate containing noncomplementary pairs is due to loop reorganization in the RuvC domain of the enzyme [32]. In addition, PAM-proximal mismatches in the DNA substrate lower enzyme efficiency because the “seed” region is involved in binding and cleavage, whereas PAM-distal mismatches have no significant impact on target DNA cleavage [29].

### 2.5. A Comparison of the Thermodynamic Parameters with the Efficiency of DNA Cleavage

There are a number of studies devoted to finding and describing the biophysical processes and patterns of the recognition and binding of target DNA by the CRISPR-Cas9 system [1,6,8,11,26,33].

The authors of Reference [11] have demonstrated that before the cleavage of a target substrate, DNA slowly dissociates from the Cas9-sgRNA complex, while no DNA dissociation from Cas9-sgRNA/DNA takes place after DNA cleavage [26]. It is known that Cas9 cleaves both strands of DNA [21], and the incisions of the two strands are not sequential events; rather, these two processes can proceed simultaneously and are limited by the DNA unfolding stage.

Thus, disturbances of double-stranded DNA structure are a factor limiting the rate of DNA substrate cleavage [11]. Liu et al. have proposed that the same stage is responsible for the specificity of such enzymes [8,11]. Our findings show a slightly different correlation. For example, for substrates S07 and S02, the melting point is lower by 1–2 °C (compared to S01), while the degree of cleavage turned out to be quite high. Substrates S04 and S08, which contain two mismatches each, were found to have the lowest melting point, but the degree of cleavage is significantly lower than that for the S01 substrate. The latter has the highest interstrand binding energy and proved to be cleaved by the enzyme the most (73%, Table 1). The degree of cleavage of substrates S07 and S02, which have the lowest interstrand binding energy, is 5–7% lower. On the other hand, substrate S09, with interstrand binding energy close to that of S01, was cleaved only by 34.5% (Table 1). Our data suggest that in the case of short duplexes with mismatches, the stages of recognition and binding of dsDNA substrates by the enzyme determines reaction rate and time rather than the thermodynamic parameters affected by the “unwinding” of DNA. We showed that the process of DNA cleavage by Cas9 endonuclease is subject to kinetic control.

## 3. Materials and Methods

### 3.1. Expression and Purification of the Cas9 Protein

Plasmid DNA transfection into *Escherichia coli* BL21 cells was performed by a standard heat shock protocol as described elsewhere [34]. The plasmid encoding Cas9 from *Streptococcus pyogenes* (spyCas9) containing a 6x-histidine tag and TEV site (pMJ806) was obtained from Addgene (Watertown, MA, USA). The Cas9 protein was prepared similarly to a protocol described previously [20], with some modifications. The protein was expressed in *E. coli* BL21 by cultivation in the LB medium supplemented with kanamycin (50 µg/mL). Protein expression was induced by the addition of isopropyl-β-D-1-thiogalactopyranoside to a final concentration of 1 mM and culturing for 18 h at 18 °C. The cells were lysed by means of a French press (process intensity was 40,000 psi). The clarified lysate was applied to a Ni-chelating HP column, and the protein was eluted via a gradient of imidazole. Elution buffer was 20 mM Tris-HCl pH 8.0, 300 mM NaCl, and 250 mM imidazole. Next, 0.5 mg of TEV protease was added per 50 mg of protein, and the sample was dialyzed in dialysis tubing with a molecular weight cutoff of 12–14 kDa against another buffer (20 mM HEPES-KOH pH 7.6, 150 mM KCl, 10% [*v*/*v*] of glycerol, 1 mM dithiothreitol [DTT], and 1 mM EDTA) at 4 °C overnight.

The eluate of the Cas9 protein was additionally purified on a Heparine HP column (GE Healthcare, Chicago, IL, USA). The bound protein was eluted by means of a gradient from 0% to 50% of a buffer (20 mM HEPES-KOH pH 7.6, 1 M KCl, 10% of glycerol [*v*/*v*], and 1 mM DTT). The concentration of the purified enzyme was determined by UV absorbance measurement at 280 nm.

### 3.2. sgRNA Preparation

The sgRNAs was prepared by in vitro transcription using T7 RNA polymerase, as described in Reference [20], and purified by the phenol–chloroform extraction method followed by isolation on adsorption columns with the help of the LRU-100-50 Kit (Biolabmix, Novosibirsk, Russia), which is similar to the mirVana™ miRNA isolation kit, and diluted with nuclease free water. Purified RNA was analyzed by gel electrophoresis and was quantified by measuring absorbance at 260 nm.

### 3.3. Oligonucleotide Substrates

All of the oligonucleotides that were tested in this study (Table 1) were synthesized in the Laboratory of Synthetic Biology at the ICBFM SB RAS on an ASM-700 automated synthesizer (BIOSSET, Novosibirsk, Russia) using the standard phosphitamide method. The products were purified by ion exchange high-performance liquid chromatography on a Nucleosil 100-5 SB column (4.6 × 250 mm) followed by reverse-phase chromatography on a Nucleosil 100-7 C18 column (4.6 × 250 mm; Macherey-Nagel GmbH, Düren, Germany). Some oligonucleotides were labeled with carboxyfluorescein (FAM). To obtain a duplex, a labeled oligonucleotide was annealed with the complementary strand in equimolar amounts. The 50 µL reaction mixture was incubated for 5 min at 95 °C and then slowly cooled down to room temperature. The resulting oligonucleotide duplex was stored at −20 °C.

### 3.4. Complex Activation

To activate the enzyme, Cas9 (1.6 µm) was preincubated for 20 min at 37 °C in a buffer containing 5% glycerol, 250 mM KCl, 0.5 mM DTT, and 10 mM HEPES-KOH (pH = 7.5), with sgRNA (1.6 µm) to assemble the complex. The volume of the reaction mixture was 30 μL.

### 3.5. Reactions with Oligonucleotide Substrates

The cleavage reaction was started by the addition of an oligonucleotide duplex (to a final concentration of 3.8 nM) to the activated Cas9-sgRNA complex. The reaction mixture was incubated at 37 °C in a buffer (20 mM HEPES pH 7.5, 125 mM KCl, 1 mM EDTA, 1 mM DTT, 6 mM MgCl_2_, and 7% of glycerol). The volume of the reaction mixture was 60 μL. Aliquots of 10 µL were taken at different time points (from 10 to 420 min). The reaction was stopped by the addition of 10 μL of a solution containing 8 M urea, TBE (9 mM Tris-NH_2_, 9 mM H_3_BO_3_, and 0.1 mM EDTA), 0.01% of bromophenol blue, and 0.01% of xylene cyanol with incubation for 5 min at 95 °C. The reaction products were analyzed by electrophoresis in a 15% polyacrylamide gel containing 8 M urea, followed by gel scanning on an Amersham Typhoon Imager (Cytiva, Marlborough, MA, USA).

### 3.6. Determination of Reaction Parameters

Data processing was carried out in Gel-Pro Analyzer software v.4 (Media Cybernetics Inc., Rockville, MD, USA). Degrees (%) of cleavage were computed from kinetic data using an exponential fit in the OriginPro software v.2015 (OriginLab Corp., Northampton, MA, USA) and are presented as an exponential curve:  y=A1×exp(−xt1)+y0, where *y* represents the degree (%) of substrate cleavage, *A*_1_ is amplitude, and *x* is time of the reaction. Error was within 10–30%.

### 3.7. Determination of Melting Temperature of Oligonucleotide Duplexes

Thermal denaturation assays of oligonucleotide duplexes were conducted as described before [15]. In brief, melting curves were recorded at a heating rate of 0.5 °C/min from 5 to 95 °C in a quartz cell with 0.2 cm light path on a Cary 300-Bio Melt spectrophotometer (Varian, Palo Alto, CA, USA) equipped with a Peltier thermostabilized multicell holder (6 × 6). Prior to melting, the samples in a buffer consisting of 10 mM sodium cacodylate, pH 6.4, 125 mM KCl, and 6 mM MgCl_2_ (similar to the reaction buffer) were heated to 95 °C, cooled, and held for 5 min at 5 °C for duplex formation. Denaturation melting curves were recorded in a multi-wavelength mode via automatic switching of the monochromator between three wavelengths (260, 270, and 300 nm) with 1 nm bandwidth. The profile of optical density at 300 nm was used for baseline correction of the thermal denaturation curves [35]. The thermodynamic parameters obtained by the fitting of denaturation curves at 260 and 270 nm were averaged. Thermodynamic parameters were calculated in MS Excel by fitting to various schemes or by a concentration method. Mass balance equations were solved using Newton’s optimization method [15].

### 3.8. Computer Simulations

#### 3.8.1. Nucleic Acid Duplex

Starting structures of oligonucleotide duplexes were generated in XLEaP (Amber20 [36]). The Amber force field bsc1 was used for dsDNA [37].

The energy of the systems was minimized in an implicit solvent model for 500 ps prior to the MD simulation. After the preparation step, the systems were minimized again, and Na^+^ (62) ions, K^+^ (41), Mg^2+^ (1), and Cl^−^ (43) ions were added to achieve conditions similar to those in the experiment. The system contained an oligonucleotide duplex, and ions were solvated with 7730 water molecules using tLEaP. An explicit water model was employed, TIP3P, under truncated “cubic” periodic boundary conditions with a distance of 8 Å. We performed a two-step process of energy minimization by steep descent in order to reduce the undesirable steric interactions and to guide dsDNA-containing systems into energetically favorable conformations.

At the first step, the solvated oligonucleotides were kept at a harmonic force constant of 25 kcal/mol/Å^2^, and the water molecules were allowed to relax. At the next step, the restriction was removed to let all the atoms move freely. Both minimization steps continued for 1000 cycles. After the energy minimization step, each system was heated gradually from 1 to 300 K for 125 ps under periodic boundary conditions. The SHAKE algorithm was used to constrain all bond lengths involving hydrogen to their equilibrium distance. The equilibration of density was performed sequentially for 50 ps and of the whole system for 500 ps without restrictions. MD equilibrium simulations of 10 ns for oligonucleotide duplexes were carried out in the NPT ensemble.

#### 3.8.2. MM-PBSA Calculations

We analyzed the obtained MD trajectories by means of molecular mechanics energies combined with the MM-GBSA in order to calculate the thermodynamic parameters of the DNA duplexes.

We generated topology files for the components of the complexes under study prior to analysis in CPPTRAJ [38]. The ionic strength of the solution was assumed to be 100 mM monovalent cations. In the calculations, each step of the MD trajectory was employed.

## 4. Conclusions

In this work, we assessed the influence of the location of a non-complementary base pair on the efficiency of the Cas9 endonuclease. We evaluated the effect of non-Watson–Crick pairs on the efficiency of DNA cleavage in terms of the influence of the structure of the formed partially complementary pairs. We also studied the impact of the location of mismatches in DNA substrates with respect to the PAM on the cleavage efficiency. Biological effects are rather difficult to describe by simple rules and regularities. The efficiency of the Cas9 system can be (and is) influenced not only by the sgRNA sequence, but also, with a high degree of probability, by the strength and correctness of the DNA duplex targeted by the genomic editing system.

We demonstrated that the presence of a non-complementary pair(s) in the “seed” sequence significantly lowers the reaction rate of DNA cleavage by Cas9. In a comparison of the efficiency of DNA cleavage with the thermodynamic parameters of the formation of nucleic acid duplexes of different complementarity, we showed that the action of Cas9 is a kinetically controlled process.

## Figures and Tables

**Table 1:** The thermodynamic parameters of duplexes’ formation and the efficiency of their cleavage.

Name	Sequence, 5′–3′	Replacement	Mismatch	ΔE, kcal/mol, 37 °C	T_m_, °C	Cleavage, %	t_1/2_, min
S01	GTGA*GTTGGACATGCTCGACATTC*GGGAGATC	-		−293.69 ± 0.08	75.2 ± 0.3	72.5 ± 1.5	18.2 ± 2.4
S02	GTGA*GTTGGACATGCTCGACATTC*GGGACATC	G→C	C:C	−281.82 ± 0.08	74.0 ± 0.3	65.8 ± 1.7	23.3 ± 4.3
S07	GTGA*GTTGGACATGCTCGACATTG*GGGAGATC	C→G	G:G	−287.03 ± 0.01	73.6 ± 0.2	67.8 ± 0.4	38.9 ± 2.2
S06	GTGA*GTTGGACATGCTCGACACTC*GGGAGATC	T→C	C:A	−290.22 ± 0.09	72.0 ± 0.2	61.9 ± 1.4	38.6 ± 7.1
S03	GTGA*GTTGGACATGCTCGACATGC*GGGAGATC	T→G	G:A	−286.33 ± 0.09	72.0 ± 0.4	56.5 ± 1.1	69.5 ± 4
S04	GTGA*GTTGGACATGCTCGAAATGC*GGGAGATC	T→G, C→A	G:A, A:G	−286.58 ± 0.11	69.7 ± 0.3	44.7 ± 2.0	43.2 ± 7.7
S05	GTGA*GTTGGACATGCTCGAAATTC*GGGAGATC	C→A	A:G	−281.59 ± 0.10	70.7 ± 0.1	41.8 ± 2.9	36.9 ± 12.5
S09	GTGA*GTTGGACATGCTCGACGTTC*GGGAGATC	A→G	G:T	−298.39 ± 0.08	73.4 ± 0.2	34.5 ± 1.7	19.6 ± 5.9
S08	GTGA*GTTGGACATGCTCGACTATC*GGGAGATC	A→T, T→A	TA:TA	−288.15 ± 0.09	70.9 ± 0.6	30.8 ± 5.6	99.3 ± 50.3

Substitutions in the sequences are highlighted in red. Sequences forming a complementary duplex are italicized. Cleavage positions are boxed off. ΔE—complex formation energy, T_m_—temperature of melting, t_1/2_—semi-cleavage time. Complexes were arranged by the combination of the percentage of cleavage and t_1/2_.

## Data Availability

Not applicable.

## Supplementary Materials

The following supporting information can be downloaded at: https://www.mdpi.com/article/10.3390/ijms232213889/s1, Figure S1: Typical melting curves of duplex recorded using different λ (UV) (260 nm and 270 nm): (a) S01, (b) S02, (c) S03, (d) S04, (e) S05, (f) S06, (g) S07, (h) S08, (i) S09; Figure S2: The cleavage assay of substrates S01 (a) and S03 (b) analyzed by denaturing 15% poly-acrylamide gel electrophoresis. Cleavage was performed using ∼3.8 nM FAM-labeled dsDNA and 32 nM Cas9-sgRNA complex (ratio 1:8).; Figure S3: The cleavage assay of substrate S01 analyzed by denaturing 15% polyacrylamide gel electrophoresis. The cleavage was performed by means of ∼3.8 nM FAM-labeled dsDNA and 800 nM Cas9-sgRNA complex (ratio 1:210).
