# Supplementary material for: Structure- and Content-Dependent Efficiency of Cas9-Assisted DNA Cleavage in Genome-Editing Systems
Source: Int J Mol Sci. 2022 Nov 11;23(22):13889. doi: 10.3390/ijms232213889 (PMC9693425; doi:10.3390/ijms232213889)
Supplement: Supplementary file 1 [file ijms-23-13889-s001.zip › ijms-1971102-supplementary.pdf]

Supplementary

Structure- and Content-Dependent Efficiency of Cas9-Assisted DNA Cleavage in Genome-Editing Systems

Svetlana V. Baranova [1], Polina V. Zhdanova [1], Alexander A. Lomzov [1,2], Vladimir V. Koval [1,2,*] and Alexander A. Chernonosov [1]

[1] Institute of Chemical Biology and Fundamental Medicine, Siberian Branch of Russian Academy of Sciences (ICBFM SB RAS), 630090 Novosibirsk, Russia
[2] Department of Natural Sciences, Novosibirsk State University, 630090 Novosibirsk, Russia
* Correspondence: koval@niboch.nsc.ru

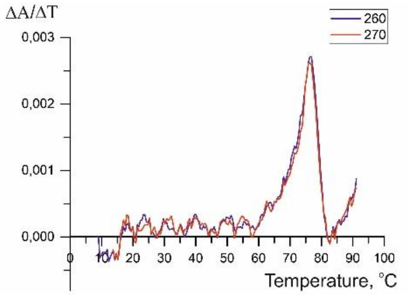

a

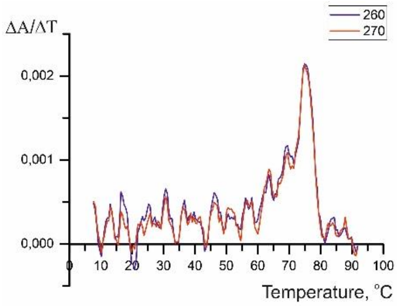

b

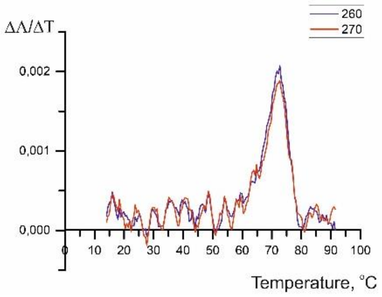

c

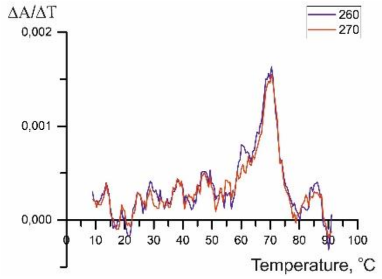

d

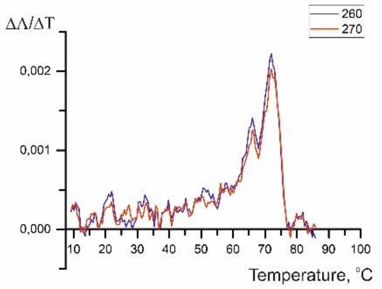

e

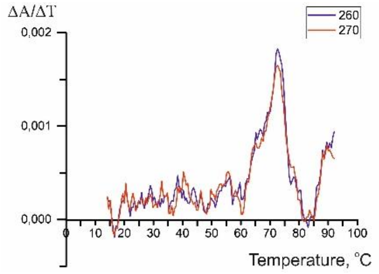

f

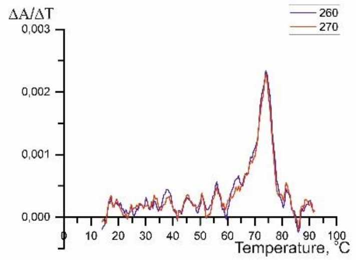

g

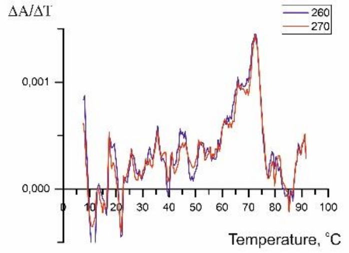

h

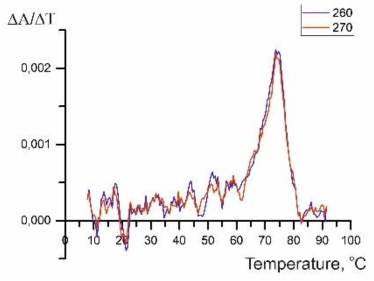

i

Figure S1. Typical melting curves of duplex recorded using different λ (UV) (260 nm and 270 nm): (a) S01, (b) S02, (c) S03, (d) S04, (e) S05, (f) S06, (g) S07, (h) S08, (i) S09.

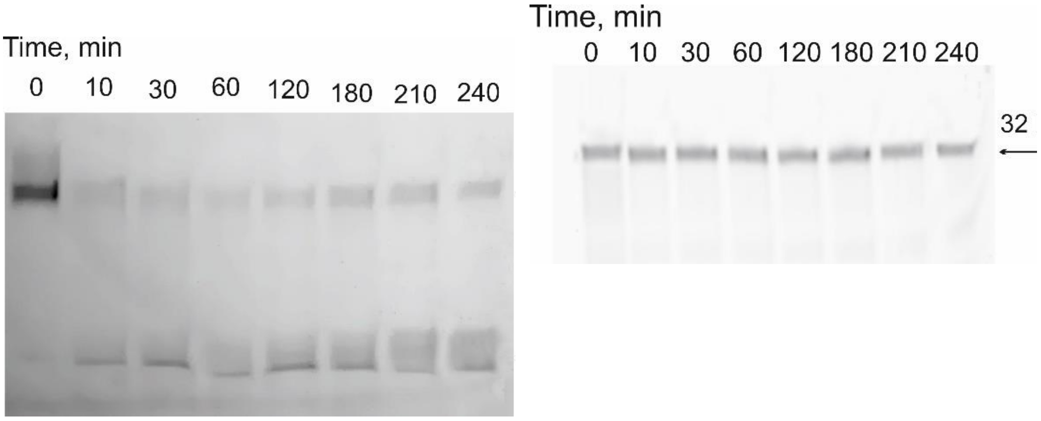

a b

Figure S2. The cleavage assay of substrates S01 (a) and S03 (b) analyzed by denaturing 15% polyacrylamide gel electrophoresis. Cleavage was performed using ~3.8 nM FAM-labeled dsDNA and 32 nM Cas9–sgRNA complex (ratio 1:8).

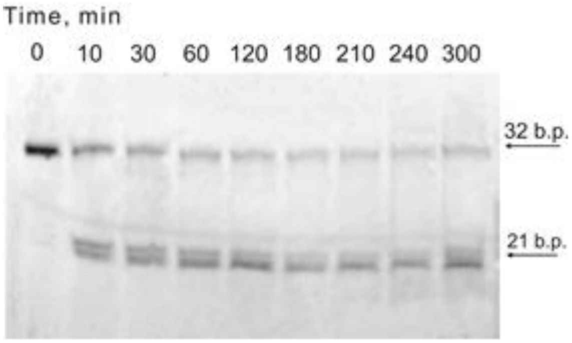

Figure S3. The cleavage assay of substrate S01 analyzed by denaturing 15% polyacrylamide gel electrophoresis. The cleavage was performed by means of ~3.8 nM FAM-labeled dsDNA and 800 nM Cas9–sgRNA complex (ratio 1:210).
